# Targeting NAD+: is it a common strategy to delay heart aging?

**DOI:** 10.1038/s41420-022-01031-3

**Published:** 2022-04-26

**Authors:** Yang Yuan, Bing Liang, Xin-Lin Liu, Wen-Jing Liu, Bing-Huan Huang, Shan-Bo Yang, Yuan-Zhen Gao, Jing-Sen Meng, Meng-Jiao Li, Ting Ye, Chuan-Zhi Wang, Xiao-Kun Hu, Dong-Ming Xing

**Affiliations:** 1 Cancer Institute of The Affiliated Hospital of Qingdao University and Qingdao Cancer Institute, Qingdao, China; 2 Interventional Medicine Center, Affiliated Hospital of Qingdao University, Qingdao, China; 3 School of Life Sciences, Tsinghua University, Beijing, China

**Keywords:** Cardiovascular diseases, Cell signalling

## Abstract

Heart aging is the main susceptible factor to coronary heart disease and significantly increases the risk of heart failure, especially when the aging heart is suffering from ischemia-reperfusion injury. Numerous studies with NAD+ supplementations have suggested its use in anti-aging treatment. However, systematic reviews regarding the overall role of NAD+ in cardiac aging are scarce. The relationship between NAD+ signaling and heart aging has yet to be clarified. This review comprehensively summarizes the current studies on the role of NAD+ signaling in delaying heart aging from the following aspects: the influence of NAD+ supplementations on the aging heart; the relationship and cross-talks between NAD+ signaling and other cardiac aging-related signaling pathways; Importantly, the therapeutic potential of targeting NAD+ in delaying heart aging will be discussed. In brief, NAD+ plays a vital role in delaying heart aging. However, the abnormalities such as altered glucose and lipid metabolism, oxidative stress, and calcium overload could also interfere with NAD+ function in the heart. Therefore, the specific physiopathology of the aging heart should be considered before applying NAD+ supplementations. We believe that this article will help augment our understanding of heart aging mechanisms. In the meantime, it provides invaluable insights into possible therapeutic strategies for preventing age-related heart diseases in clinical settings.

## Facts

- NAD+ deficiency is the primary inducement to heart aging, resulting in decreased energy synthesis. The heart manifests as thinning of the ventricular wall and enlargement of the cardiac chamber diameter, which leads to heart failure with the continuation of the process.
- As an anti-aging target, NAD+ also plays an important regulatory role in the heart. NAD+ mediated deacetylation is widely involved in regulating transcriptional signals for cardiomyocytes protection and interacts with ROS and calcium signals.
- NAD+ mediated mitochondrial quality control is the core mechanism of preventing heart aging, involving mitochondrial dynamics, permeability, biological synthesis, and mitophagy. As a coenzyme, NAD+ also further stabilizes mitochondrial oxidative phosphorylation.

## Open questions

- Considering the characteristics of the heart as a blood-pumping organ, do more intracellular factors need to be considered when targeting NAD+ signals?
- Do comorbidities of the aging heart affect the protective effect of NAD+ ?
- Do different forms of NAD+ precursor intervention affect the protective effect of NAD+ ?

## Introduction

Aging is a gradual, continuous, naturally occurring process. It is closely related to the development of several common chronic diseases, such as type II diabetes, hypertension, neurodegenerative diseases, etc. [1, 2], which are manifested by the decline and failure of multiple organ functions [1]. Among them, heart failure caused by heart aging at the cellular level is a high-risk factor affecting the life span of an individual [3]. Cardiovascular drugs commonly used in the treatment cannot inhibit the development of the disease effectively; instead, they only play a partial role in reducing the symptoms. It is necessary to find novel approaches for preventing heart aging by targeting the endogenous signaling pathways in cardiomyocytes. However, the investigation of regulatory mechanisms in the heart is still insufficient. Many in vivo and in vitro studies showed the importance of NAD+-dependent Sirtuins (SIRTs) deacetylation activity in the anti-aging process [4, 5]. However, discussing a single mechanism may neglect other targets of auxiliary intervention since the function of NAD+ itself is also an important part of the mitochondrial respiratory chain, wherein it interacts with most of the intracellular molecules. Recently, it has been believed that the depletion of NAD+ with aging is associated with oxidative stress injury, which is a crucial factor of aging, and NAD+ deficiency has a certain degree of duality for myocardial protection. Whether NAD+ signaling is the key variable affecting other cellular signaling pathways needs to be further investigated. A recent FDA-approved anti-aging supplement, nicotinamide mononucleotide (NMN), regulates NAD+ signaling to achieve cell self-regulation and improves adaptation to the environment of the aging cell from the perspectives of regulating aging-related gene transcription and inhibiting aging-induced stress imbalance [6]. However, whether a large amount of NAD+ supplementations would definitely induce heart anti-aging effect is also controversial. Therefore, this article focuses on the mechanism of NAD+ signaling in cardiomyocytes and various intracellular senescence processes, trying to understand its impact on several cell endogenous processes such as inflammation, apoptosis, autophagy, mitochondrial damage, etc. We aim to identify more feasible research directions of NAD+, resolve the existing unknown areas, and provide novel strategies and inspiration for our related research.

## Aging heart and associated mechanisms

### Implications of NAD+ in pathophysiological processes driving heart aging

**Fig. 1:**
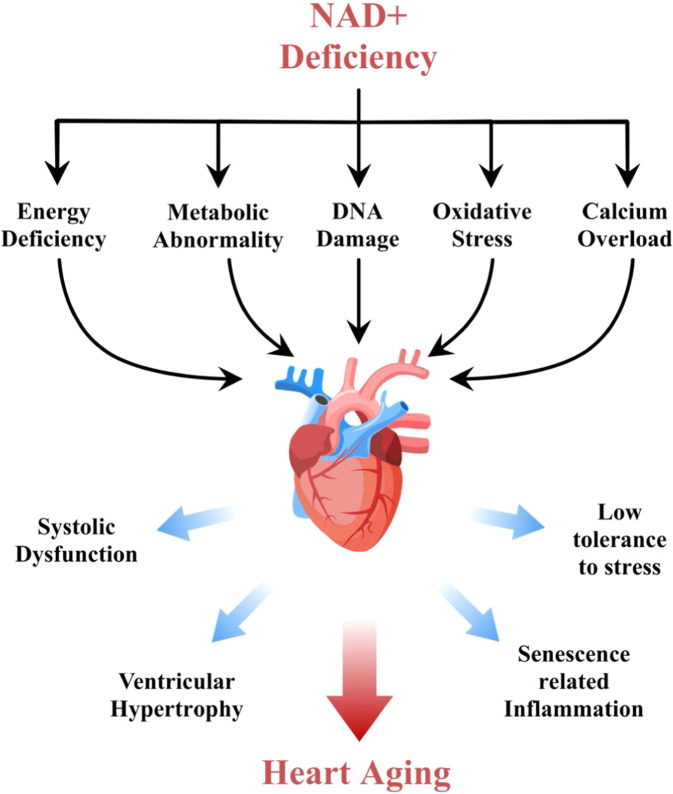
Relationship between NAD+ deficiency and heart aging. The lack of NAD+ in the aging heart could play a crucial role in causing various changes in different cellular pathways involved in cardiac damage. The outcomes of NAD+ deficiency include decreased heart function and increased ventricular dilation risks in inflammation and stress, all of which are well known to speed up the process of heart aging.

The aging heart is accompanied by decreased energy synthesis and functional levels, such as low ejection fraction, shortening fraction, and increased left ventricular diameter, causing dilated cardiomyopathy and insufficient blood supply, which are the main manifestations of heart failure [1, 7]. Myocardial aging is also a high-risk factor for malignant arrhythmia and atrial fibrillation [8]. DNA damage, inflammatory reaction, and abnormal lipid metabolism could be well-recognized pathological characteristics of the aging myocardium [9]. Notably, a lack of NAD+ in the cardiomyocytes may cause hypoxia, which is considered as one of the leading factors for aging [10]. At the organelle level, genetic evidence showed that the change in mitochondrial permeability caused by myocardial aging is the main reason for the increase in cardiac mechanical stress sensitivity. Inhibition in the rise in mitochondrial permeability may help to reduce the decline in cardiac function caused by heart failure [11]. It is suggested that the decrease in ATP synthesis is also an important cause and an inducer of heart failure in the aging organisms [12]. A comparative study of the effects of glucose uptake on aging-induced myocardial mitochondria damage showed that the difference in energy intake could not alter the fact that aging causes damage to myocardial mitochondria [13, 14]. NAD+ and its downstream signals may be the key regulatory factors of energy utilization, which are associated with age and gender difference. However, an exploration of goNAD+al hormone regulation in cardiac aging is still lacking. In general, the metabolic abnormality is the first step to be considered in NAD+-deficiency-induced aging [15]. Low aerobic energy supply and high levels of glycolysis are the main reasons for the aging of most tissues or organs. Studies have shown that after mitochondrial irreversible and complete aging, a higher NAD+/NADH ratio helps reduce glycolysis, avoiding the abnormal metabolism of tryptophan due to lysosome-associated membrane protein 2 (LAMP-2) deficiency [16]. Furthermore, some studies suggested that the decrease in NAD+ with aging is closely related to mitochondrial permeability transition pore (mPTP) opening in cardiovascular stress. These results indicate that the reduction in mitochondrial NAD+ preservation may be a substantial cardiomyocyte senescence-inducing factor, manifested by the decrease in adaptability to adverse homeostasis changes, such as high glucose, hypoxia, drug stress, or ischemia–reperfusion (I/R) injury [7]. The mitochondrial repair mechanism is further declined by reducing mitochondrial oxygen consumption efficiency, thus forming a vicious circle [17]. Figure 1 is a schematic diagram showing the relationship between NAD+ deficiency and heart aging (Fig. 1).

### Implicated mechanisms of the anti-aging effect of NAD+

Due to mitochondrial damage, excessive accumulation of reactive oxygen species (ROS), mainly stress-induced H_2_O_2_, can cause membrane lipid peroxidation and further damage to cellular structures, including cytomembrane, mitochondrial membrane endoplasmic reticulum, etc. The aging heart is also accompanied by an overall decrease in antioxidant activities of enzymes, such as manganese-dependent superoxide dismutase (MnSOD), or a direct injury to DNA and mtDNA, which lead to high expression of apoptosis factors [12]. Oxidative stress injury is a common feature in the process of aging in most tissues and organs, including the heart [9]. The accompanying DNA damage is also an essential factor of myocardial aging and is commonly associated with aging-induced inflammatory response (senescence-associated secretory phenotype, SASP) [18]. For example, the cluster of differentiation 38 (CD38) and tumor necrosis factor (TNF-α) downregulated NAD+ through competitive binding of NAD+ precursor, which interfered with the synthesis of NAD+ by enzymes [19]. Knockdown of NAD+ hydrolase, a CD38 ectoenzyme, effectively inhibited D-gal-induced cardiomyocyte senescence and significantly reduced the senescence markers p16 and p26, leading to a prolonged life span of cardiomyocytes [20]. In particular, a similar phenomenon was also manifested in chemotherapeutic drug-induced cardiomyocyte toxicity, which has a certain association with senescence properties. For instance, chronic doxorubicin (DOX) intervention can also lead to cardiomyocyte aging, suggesting that the aging mechanism shares many irreversible stress factors with the cardiac injury pathway, such as decreased *Sirtuins* expression, increased p38 mitogen-activated protein kinases (p38MAPK) signaling, and decreased antioxidant enzyme levels represented by MnSOD [21, 22].

From the perspective of molecular mechanism in aging cardiomyocytes, the reduction in self-regulation and adaptability or direct damage to DNA and mtDNA, promotes an increase in oxidative stress and apoptosis and induces high expression of pro-inflammatory cytokines, such as nuclear factor kappa-light-chain-enhancer of activated B (NF-κB). In this way, more exogenous stress factors will accelerate the aging process, forming a vicious circle. On the other hand, NF-κB is also directly and positively regulated by Clock Circadian Regulator (*CLOCK*) genes. In the perspective of protective mechanisms of aging cardiomyocytes, inhibition of BMAL1/Clock by NAD+ reduced NF-κB binding to DNA, which further decreased the formation of pro-inflammatory mediators and improved mitochondrial function [23]. In addition, the biological rhythm of the cardiovascular system also involves the regulation of PPARγ. The downstream target gene *REV-ERB* of PPARγ is one of the core CLOCK components [24]. Moreover, PPAR is closely related to the expression and activity of antioxidant enzymes [25]. Therefore, the aging process could be driven by various cellular mechanisms within the organism [26].

## NAD+ synthesis and metabolism in the aging heart

### NAD+ synthesis

NAD+ is a crucial coenzyme in mitochondrial oxidative phosphorylation (OXPHOS), and it is the oxidized form of NADH of complex I. NAD+ acts as a major hydrogen carrier and plays a vital role in the electron transport chain. In other words, NAD+ can also be considered to be consumed by the TCA cycle but to regenerate from NADH by electron transport chain [27]. Recent studies have shown that similar to ROS, NAD+ acts as an intracellular second messenger and may establish mitochondrial protection through the NAD+/Sirt pathway [28]. NAD+ is mainly synthesized from NAM by the rate-limiting enzyme Nam phosphoribosyltransferase (NAMPT) and degraded to Nam by NAD+-degrading enzymes in mammals [29]. NAD+ can also be re-synthesized from nicotinic acid (NA) and nicotinic acid riboside (NAR) using nicotinate phosphoribosyltransferase and NAM riboside kinase ½ (NRK1/2), respectively. However, most tissues rely on amidated synthetic forms. NRKs and NAMPT can recover nicotinamide riboside (NR) and NAM into NMN, further converted into NAD+ by NMNAT eventually [30, 31]. Studies have shown that the aging-related *WRN* gene regulates the transcription of the critical NAD+ biosynthetase NMN adenyltransferase 1 (NMNAT1) [32]. Loss of the *NAMPT* gene also affects the survival in adult mammals [33]. NAD+ improves the mitochondrial quality by restoring the metabolic environment of intracellular NAD+ by DAF-16/FOXO controlled, germline tumor affecting-1 (DCT-1-) and Unc-51 like autophagy activating kinase 1(ULK-1)-dependent mitophagy. With aging, NAMPT has specific compensatory properties related to the upregulation of CD38 in decomposed NAD+ [34]. However, NAD+ supplementation also induces the synthesis of NAMPT, promotes a virtuous cycle of NAD+ synthesis, and contributes to anti-aging [34]. Some studies believe that the expression levels of NAMPT and NRK2 have little effect on the cellular NAD+ content but may cause the maladaptation of aging myocardium during endurance exercise or work overload [29, 35]. In humans, short-term 3-day NAD+ supplementations, such as NMN, NAM, NAR, and other NAD+ precursors, can produce mitochondrial conservation effects and play a key role in regulating redox status and energy metabolism, thereby inhibiting heart failure [36]. NAD+ depletion is one of the significant pathogeneses of heart and kidney diseases, and NAD+ supplementation shows therapeutic potential as an approach to restore metabolic levels and physiological functions. In particular, NAD+ plays an auxiliary substrate function in the deacylation reaction of the SIRTs family. Therefore, enhancing SIRT activity by NAD+ supplementation may be viable for treating heart disease [28].

### NAD+ and energy synthesis

**Fig. 2:**
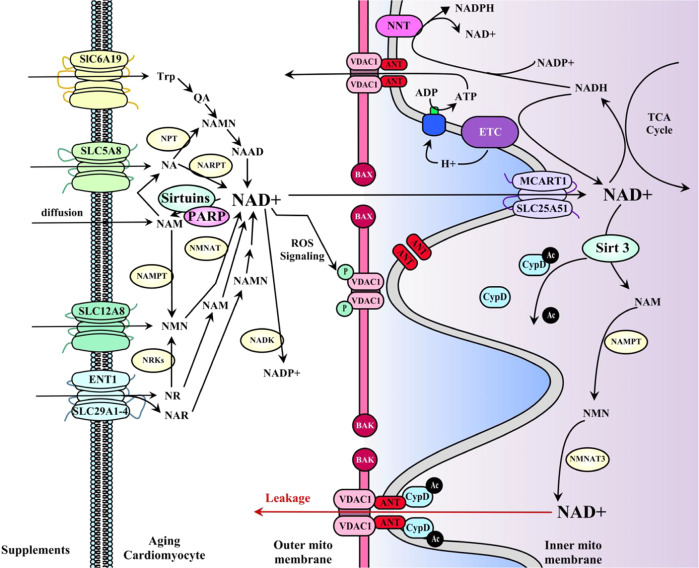
Schematic diagram illustrating intracellular pathways of NAD+ synthesis, NAD+ metabolism, and NAD+ signaling pathway in mitochondria. NAD+ can be synthesized from multiple precursors, including Trp, NA, NAM, NMN, NAR, and NR. NAD+ mainly exerts biological functions in mitochondria via regulating mitochondria membrane transporter SLC25A51 (MCART1 in the heart), affecting the transport of metabolic substrates, ATP, and ions. Sirtuins and PARP are the main NAD+ consumers.

NAD+ supplementation showed a specific anti-apoptosis ability in cardiomyocytes. The preserved NAD+ in the mitochondrial matrix could inhibit mitochondrial damage and induce the release of cytochrome C (Cyt C) [23, 37]. mPTP is a voltage-gated ion channel across mitochondrial inner and outer membranes and is closely linked to mitochondrial damage. It is directed by voltage-gated ion channel (VDAC), adenine nucleotide translocase (ANT), F_1_F_0_ATP synthase, cyclophilin D (CypD), cyclosporin A (CsA), and transmembrane protein complexes composed of other subunits. Supplementation of NAD+ through the SIRT3 signal acts on the binding of CypD to intimal ANT, which helps to delay the opening of mPTP, thus protecting the mitochondria [36]. CypD knockout (KO) in elderly mice showed better anti-aging effects, such as inhibition of mitochondrial swelling and a higher NAD+/NADH ratio, but further exploration is needed to explain the exact mechanism in heart tissue [38]. Moreover, the aerobic oxidation of acetyl-CoA in mitochondria is affected by NADH and succinic acid [39]. In the mitochondrial inner membrane, the free penetration of phosphopyruvate (PEP), an intermediate product of glucose metabolism, can inhibit the oxidation of NADH, improving the dehydrogenation efficiency of succinic acid, and promote the oxidative phosphorylation reaction, suggesting that the supplement of energy synthesis substrate is beneficial for the preservation of NADH in cells. Additionally, improving glucose utilization efficiency helps reduce the myocardial inflammatory response and inhibit injury-induced myocardial fibrosis [40]. However, some studies on cardiac I/R injury suggest that excessive NADH supplementation may induce more superoxide production and aggravate oxidative stress injury [41]. Excessive NADH may promote anaerobic glycolysis that potentially accelerates the aging process [27, 42]. Therefore, the abnormalities such as altered glucose and lipid metabolism, oxidative stress, and calcium overload could interfere with NAD+ function in the heart. And specific physiopathology of the aging heart should be considered before applying NAD+ supplementations [43]. Moreover, NAD+ supplementation can also be considered to use as a substrate to promote PARP-mediated DNA repair for promoting myocardial anti-aging effect [40]. A conclusive diagram illustrating the intracellular pathways of NAD+ synthesis and metabolism and the NAD+ signaling pathways in mitochondria of the aging heart is in Fig. 2.

## Molecular mechanisms underlying the anti-aging effect of NAD+

### Cross-talk with pathways inside the nucleus

As mentioned earlier, the low level of energy synthesis and unbalanced oxidative stress are important initial links, which lead to the increased expression of pre-apoptotic signals and inflammatory factors. Therefore, the downstream regulatory factors, such as 5 ′ AMP-activated protein kinase (AMPK), which are sensitive to ATP energy levels, play a vital role in the inductive mechanism. Studies have shown that AMPKα2 was highly related to the biological rhythm of cardiomyocytes, in which AMPKα2 had a more apparent inductive effect [44]. The regulation of senescence by AMPK mainly links to the SIRTs family, and the intracellular signals generated by AMPK are important core substances that inhibit the senescence of cardiomyocytes. In Sirt1-deficient mice, the aging heart showed reduced tolerance to ischemic stress, associated with decreased ATP synthesis and increased mitochondrial ROS production [45]. In the absence of NAD+, reduced deacetylation of NF-κB by Sirt1 initiated the transcription of pre-apoptotic factor NLPR3, which ultimately led to the increase in inflammasome and the induction of age-related inflammatory factors expression [23]. *Sirt1* gene KO decreased ejection fraction, increased oxidative stress, and increased endoplasmic reticulum stress in the aging mice [46]. The binding of ATP to the C-terminal of Sirt1 might limit its activity and inhibit its anti-stress damaging effect [47]. Moreover, it has been shown that AMPKα2 deficiency inhibited NAD+ synthesis, the expression of the Sirt1/PGC-1α pathway, and affected the repairability of cardiomyocytes [44]. Recent reports suggested that Sirt1-mediated NK-κB inhibitory regulation was also depend on the effect of *Sirt6* gene silencing on DNA repair targeting H3K56Ac, but information on myocardial tissues is still lacking [48]. In NAD+-dependent deacetylase Sirtuin-3 (Sirt3-/-) mice, the absence of Sirt3 caused increased sensitivity to Ca^2+^ in cardiomyocytes, resulting in swelling of mitochondria, which ultimately affected the life span of the mice and accelerated the senescence of cardiomyocytes [11].

It is well known that regulation of cardiomyocyte protein deacetylation is involved in a wide range of interactions between DNA transcription and histone modification, including those of the *Sirtuins* gene itself, and is affected by NAD+ levels and its related salvage pathway [17]. *Sirt2* expressed in the cytosol has recently been considered to be involved in the anti-aging mechanism of the heart. Using a serum deprivation test, the increase in *Sirt2* gene transcription level was regulated by the upstream RhoA/SRF signal through its SRF binding site, inducing feedback on energy metabolism, stress, toxic metabolism, etc [49]. RhoA/SRF signaling inhibitor CCG-1432 canceled the *Sirt2* induction [49]. Recently, *Sirt7* has also been reported to have tissue-specific anti-aging effects in the myocardium, which may be associated with deacetylated activations of *Sirt1* and *Sirt2* [49–51]. The FOXOs family is a class of nuclear transcription factors regulated by upstream Akt/PI3K cardiac protective signals. FOXO1, FOXO3, and FOXO4 have been shown to exert anti-aging effects in the organism, and FOXOs-assisted protective expression was unaffected in the transverse aortic constriction (TAC) model with MnSOD deficiency [22]. Upregulation of *Sirt* induced deacetylation of downstream *FOXO* genes and inhibition of NAD+ synthesis caused downregulation of the Sirt3/FOXO4 pathway, which was consistent with inhibited TCA cycle and ATP synthesis, thus affecting the survival of mice [32]. Inhibition of NAD+ also resulted in increased acetylation of *SOD2*, *PGC-1α*, *GCN5*, and *p65*; decreased activity of protective enzymes; and increased aging-related heart damage [34]. Another critical factor, the STAT protein family, the signal transducer and activator of transcription, consists of seven species (*STAT-1, STAT-2, STAT-3, STAT-4, STAT-5a, STAT-5b*, and *STAT-6*), all of which are expressed in the heart. STAT are tyrosine kinases that can be activated by Janus kinase (JAK), causing their translocation into the nucleus to form dimers or heterodimers, resulting in the regulation of gene transcription. The JAK/STAT pathway activation plays a vital role in expressing stress response genes. Moreover, the activation of *STAT1* is related to cell apoptosis. However, it can be inhibited by Sirt1 [43]. On the contrary, the activation of mitochondrial *STAT-3* inhibited mPTP and thus contributed to cardiac protection [52, 53]. The localization of STAT proteins in the heart is not limited to cytosol and nucleus. Recent studies have shown that *STAT* was expressed in the mitochondria of cardiomyocytes [54]. Most studies of STAT proteins in cardiac function have focused their roles in cell apoptosis, heart failure, hypertrophy, and I/R damage [55]. To better understand the mechanisms underlying heart aging, the role of STATs in aging hearts should be further investigated.

### Cross-talk with ROS signaling

IGF-1/insulin signaling is necessary for prolonging the life of cardiomyocytes [56]. Activation of downstream signals, Akt/PI3K, and mechanistic target of rapamycin (mTOR) can further induce autophagy and other cytoprotective mechanisms, which helps to provide a stable metabolic environment and balance the oxidative stress. These signals interact with NAD+ signaling, which affects the regulation of survival-related nuclear transcription signals [57]. Recently reported, deacetylation based on NAD+ signaling was able to enhance the expression of key proteins implicated in PI3KIII-dependent autophagy or promoted Parkin-independent mitophagy through the liver kinase B1 (LKB1)/AMPK/tuberous sclerosis complex 2 (TSC2) or the SIRT3/FUNDC1 pathways [9]. The imbalance in oxidative stress regulation is the significant consequence of ROS production, which further affects the life span of an organism. Recent studies have shown that ROS can also induce cell apoptosis through multiple intracellular signal mediation, thus accelerating cell aging [40]. Conversely, a moderately high level of ROS is necessary for myocardial protection, which is achieved by inducing protective signals [58, 59]. Therefore, the NAD+ signal may be an essential rebalancing factor of ROS signal [33].

NADH dehydrogenase, a complex I protein, transfers electrons from NADH to Flavine MonoNucleotide (FMN), FES centers, and ubiquinone. Under abnormal mitochondrial membrane potentials, these pathways easily generate ROS [39]. ROS regulates cardiac contractility at multiple levels. Due to the direct modification of ion channels and transporters and the changes in intracellular contraction signals. Overactive NAD(P)H oxidase (Nox), xanthine oxidase, or excessive ROS generated by mitochondria are considered to play a leading role in cardiomyopathy [60]. Furthermore, in MnSOD ± mice, the expression of *Nox1* did not change, suggesting that the factors that cause an increase in ROS are not the determinants of *Nox1* expression. The key enzyme causing the ROS-induced ROS generation (RIRG) phenomenon may be *Nox4*, responsible for accelerated aging in MnSOD-deficient mice [22]. The abnormal cardiac activity begins with rapid activation of voltage-gated sodium channels (such as Nav1.5 and voltage-dependent L-type calcium channel (LTCC)) to propagate action potentials. ROS-dominated oxidative stress is thought to affect the life span of organisms [61]. Simultaneously, ROS activates intracellular REDOX signals, such as proto-oncogene tyrosine-protein kinase Src (c-Src), Protein tyrosine kinase 2 (Pyk2), extracellular signal-regulated kinases (ERKs) 1/2 (ERK1/2), and big mitogen-activated protein kinase 1 (BmK1) [61]. These factors affect the expression of proteins in NAD+ signaling by varying degrees.

Nrf2 is a vital gene transcription factor that regulates antioxidation and redox reactions. However, it is expressed at low levels during the aging process [22]. Nrf2 deficiency can lead to dysregulation of cardiomyocytes under oxidative stress and increased sympathetic excitability [62, 63]. Therefore, applying dietary restrictions or implementing some drug interventions can induce the regulation of Nrf2/ARE and increase the expression of antioxidant enzymes, such as GSH. This process is influenced by Kelch-like ECH-associated protein 1 (KEAP-1), a cytoplasmic inhibitory protein. KEAP-1 restricts *GSH* gene expression by forming a complex through Nrf2 [64]. In addition, the activation of ARE induces the activation of NAD(P)H quinone oxidoreductase-1 (NQO1) and heme oxygenase-1 (HMOX1), which further enhances the antioxidant capacity [65]. Furthermore, the high expression of γ-glutamylcysteine synthetase catalytic (GCLC) through the Nrf2 signal helps inhibit the aging and apoptosis induced by high glucose intake in myocardial cells [48]. PGC-1α, an inducer of Nrf2, is another crucial substance that promotes the synthesis of antioxidant enzymes. The lack of PGC-1α in the kidney causes a decrease in NAD+ synthesis and affects TFEB-dependent lysosome function [66]. PGC-1 is regulated by the upstream NAD+/AMPK signal, which provides cardiac protection by phosphorylating TSC2 to inhibit mTOR [9]. Therefore, NAD+ is the crucial bridge between energy metabolism and oxidative stress regulation [45].

### Cross-stalk with calcium signaling

**Fig. 3:**
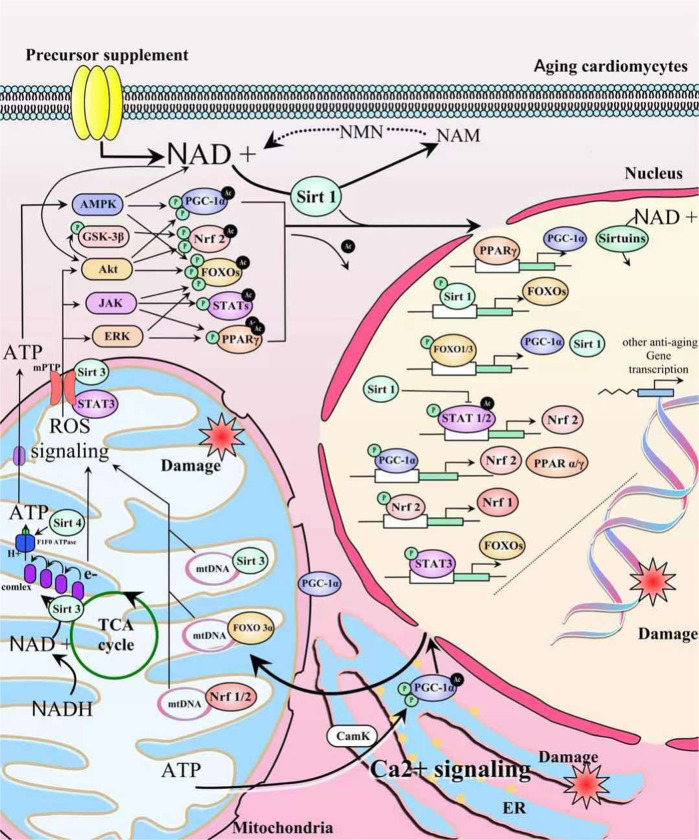
Schematic diagram showing the cross-talks between NAD+ and intracellular pathways in mitochondria and nucleus. In cardiomyocytes, NAD+ regulates a variety of nuclear transcription regulatory proteins such as Sirts, FOXOs, and STATs through its induction of deacetylation. Its extensive regulatory capacity is not limited to the cytoplasm but also in mitochondria and inflammasomes. The initial factor may be related to AMPK and ROS signaling.

**Fig. 4:**
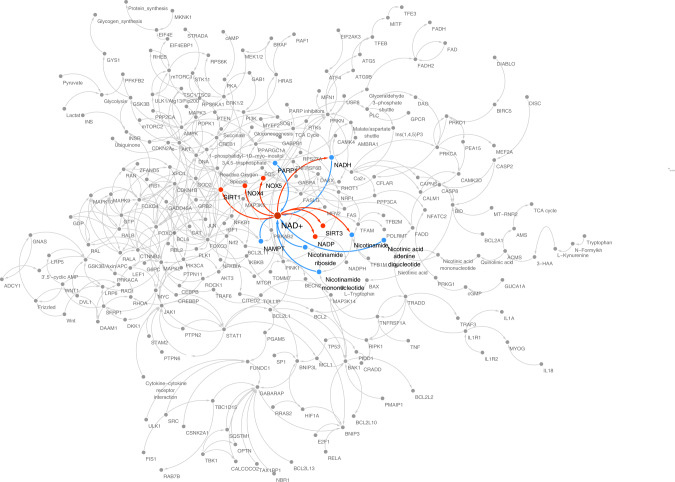
Protein–protein interaction networks between NAD+ and associated regulatory proteins implicated in heart aging. The presented network is drawn by the JAVA-based platform Cytoscape 3.8.2 (http://cytoscape.org): the protein-protein interaction (PPI) data were merged by multiple databases of Kyoto Encyclopedia of Genes and Genomes (KEGG), Wiki pathway, NDEx, PSICQUIC, and String. At first, the data covering the overlap part of cardiac hypertrophy and aging pathway were collected, which were then merged with the NAD+-related pathway. The disconnected nodes have been removed. The blue nodes and lines indicate direct upstream factors of NAD+. The red nodes and lines indicate the direct outcome of NAD+. NAD+, placed in the center of the network, is associated with several regulatory proteins, especially the Sirtuins, FOXOs, and STATs families, and the oxidative stress-related pathways implicated in heart aging.

Poor calcium storage capacity of the myocardium, which is the main reason for the decline in myocardial function during aging, is related to poor calcium transport [67]. NAD+ signal interacts with Ca^2+^ signal in myocardium [40]. Repeated apnea and intermittent hypoxia cause NAD(P)H oxidase (Nox)-dependent ROS production, wherein most of the ROS are superoxide anion that further activates phospholipase C, resulting in the activation of calmodulin kinase C (CaMK) and protein kinase C [8, 68]. It has been shown that NR as an exogenous supplement could promote CaMKII-mediated RyR2 phosphorylation in the diastolic phase by increasing NAMPT/NAD+ and promoting calcium release from the Sarcoplasmic Reticulum (SR) [8]. Due to the decrease in stress resistance caused by the aging of the myocardium, the sodium-calcium exchanger (NCX) was reversed under the action of stress factors, resulting in intracellular calcium overload [69]. The sensitivity of mitochondrial NADH reductase and ERK pathway to ROS stress decreased Na+/H+ antiporter *(NHX*) expression [70]. The downregulation of *NHX* possessed practical significance in inhibiting the increase in pH and calcium overload caused by Na^+^ influx and H^+^ outflow during the recovery process, which helps avoid the vicious circle of oxidative stress caused by the high concentration of intracellular Na^+^. Some studies have also shown that inhibition of CD38 contributed to saving NAD+ and reduced calcium overload through inhibition of Ca^2+^ mobilization [71]. These hypotheses may serve as a series of intervention methods, aiming to enhance the regulation of NAD+-related protective signals. In addition, more protective signals might be beneficial for delaying myocardial aging and improving tolerance. For example, NMN as a precursor supplement contributes to the formation of Sirt7-dependent cardioprotection, which involves the deacetylation of GATA4 and the interaction with TGF-β1 signal [72]. Increased NAD+ deacetylation could inhibit the protein kinase R-like endoplasmic reticulum kinase (PERK)/eukaryotic translation initiation factor 2 pathway and reduce endoplasmic reticulum stress [73]. Moreover, it could also promote an adaptation of the aging myocardium to hypoxia by activating the HIF-1 signal and NO signal [10, 74]. This evidence strengthens the associations among NAD+, ROS, and calcium. Figure 3 shows the cross-talks between NAD+ signaling and other intracellular pathways such as ROS and Ca2+ signaling in mitochondria and different transcription factors in the nucleus. Besides, a protein-protein interaction network (Fig. 4) illustrates the interactions between NAD+ and main regulatory proteins in pathways implicated in aging hearts.

## NAD+ regulates mitochondrial homeostasis in the aging heart

### Mitochondrial dysfunction and mitochondrial quality control

Mitochondrial quality control coordinates various processes (proteostasis, biogenesis, dynamics, and mitophagy) to maintain cell homeostasis. Mitochondrial dysfunction, amplified by failing quality control processes, is believed to be a major mechanism underlying cardiac aging and cardiovascular disease. There is a reduction in the ability of mitochondria to produce NAD+ in aging cardiomyocytes, which is accompanied by mitochondrial DNA damage [11]. Increased expression of uncoupling proteins (UCP) promotes aging-associated mitochondrial dysfunction in the heart [45, 75]. UCP also acts on the abnormal calcium transport between the endoplasmic reticulum and mitochondria, causing mitochondrial calcium overload through RyR2 and VDAC, which leads to weakened stress tolerance [67]. Inhibition of mPTP opening contributes to preserving NADH and ATP, induces a virtuous cycle of NAD+ synthesis, and delays mitochondrial depolarization [34]. In aging rats, NMN supplementation reduced the increase in mitochondrial membrane potential (ΔΨm) when undergoing subsequent I/R stress [76]. Mitochondrial depolarization induces various endogenous mitochondrial protective mechanisms, such as the regulation of mitophagy and mPTP pores. A recent study showed that Parkin-induced mitophagy might be an essential mechanism for inhibiting myocardial aging [77]. In the heart, PINK1 is upstream of parkin. PINK1/Parkin-dependent mitophagy pathway depends on the macroautophagy protein level and the mitochondrial outer membrane protein guidance. The transcriptions of both *PINK1* and *Parkin* are associated with NAD+-mediated nucleus signaling, for example, Nrf1/2 [78]. Parkin, as an E3 ubiquitin ligase, mediates the ubiquitination of VDAC1, where parkin can be recruited by PINK1 from the cytoplasm to the outer membrane of mitochondria with decreased ΔΨm, and p62 can be further recruited to the mitochondria to induce the autophagosomal membrane to enclose the mitochondria and initiates mitophagy [79]. Hence, damaged mitochondria can be selectively scavenged by mitophagy. Parkin also ubiquitinates the mitochondrial fusion protein Mitofusin 1 and 2 (mfn1/2), thereby promoting the repair of damaged mitochondria by regulating mitochondrial dynamics [80]. Phosphorylation of serine/threonine kinase PINK1 activates parkin and induces its translocation from cytoplasm to mitochondria, which contributes to the anti-aging effect in the heart [81]. Tom70 protein is a component of the translocase of mitochondrial outer membrane (TOM) complex. It may be the initial protein recognized by precursor proteins of parkin and p62. TOM was found to guide parkin and p62 to locate on mitochondrial membrane and promote mitophagy and was reported to inhibit pathological cardiac hypertrophy [82].

An exogenous injection of uncoupling agent carbonyl cyanide-p-trifluoromethoxyphenylhydrazone (FCCP) resulted in proton gradient reduction, blockage oxidative phosphorylation F_1_F_0_ ATPase coupling, mitochondrial membrane depolarization, all of which are associated with cardiomyocytes aging [83]. However, FCCP restores NAD+ because of inhibited OXPHOS [27]. It was found that exogenous FCCP enhanced the transport of parkin to mitochondrial membrane [79], suggesting that mitochondrial membrane depolarization is the chief factor in inducing mitophagy. In addition, a BH-3-specific domain protein also mediated parkin [84]. These proteins may also be affected by senescence-related apoptotic signals [85]. For instance, Bcl-2 and Bcl-xl acted as anti-apoptotic proteins by binding to BH-3 protein, and NAD+ enhanced their expression through phosphorylation of Akt and STAT1 signal [86, 87]. At the mitochondrial outer membrane, BCL2/adenovirus E1B 19 kDa protein-interacting protein 3 (Bnip3) and NIX interact with microtubule-associated proteins 1A/1B light chain 3 (LC3) of the autophagosome, which not only mediated outer membrane permeability but also acted as a mitophagy initiation signal [88]. However, it has been reported that the upregulation of mitophagy mediated by parkin might be related to mitochondrial damage in cardiac stem cells (human cardiac progenitor cells/hCPCs). In contrast, the mitophagy mediated by Bnip3 and NIX would be more closely related to the aging mechanism [74]. Bnip3 promotes mitochondrial membrane depolarization by directly opening mPTP. mPTP opening, Ca^2+^, and ROS also reversely induce Bnip3, thereby upregulating the level of mitophagy [89]. However, it has been reported that a low Sirt1 level during aging could induce increased expression of *Bnip3* in the heart, which might be related to the reduced induction of reparative mitophagy, which further caused the opening of the outer membrane pore and aggravated mitochondrial damage [45]. Moreover, Bnip3 induced mitochondrial Bax, which further affected the TOM complex through mitochondrial transmembrane Bim [90]. Therefore, Bnip3 might mediate parkin translocation to mitochondria through the Tom protein complex. In addition, NIX is bound to LC3 through GABA type A receptor-associated protein (GABARAP) for initiation in NIX-dependent mitophagy. However, the mitophagy mediated by NIX might not be related to depolarization but cardiac hypoxia [74]. Furthermore, it is suggested that overexpressed BH-3-mediated mitophagy and autophagy might give rise to autophagic exhaustion, leading to speeding up of heart aging process [91].

### Mitochondria-related apoptosis and its possible regulation

The regulation of autophagy by intracellular ROS level involves tumor suppressor protein p53 and TP53-induced glycolysis and apoptosis regulator (TIGAR) [92]. TIGAR is a class of fructose-2,6-diphosphate, which reorients the metabolic intermediate of glucose decomposition to the oxidative branch of the pentose phosphate pathway. P53 regulates TIGAR, which is associated with decreased NAD+/NADH redox couple [93, 94]. Recently, TIGAR was found to aggravate LV dysfunction, fibrosis, and oxidative stress in a pressure-overload heart failure model [95]. Normal TIGAR upregulated the production of NADPH, thereby reducing the level of ROS in cells and the sensitivity of cells to apoptosis-related oxidative stress [96]. Studies have shown that p53/TIGAR mediates the inhibition of mitophagy. In p53 and TIGAR KO mice hearts, an increase in ROS involves the activation of Bnip3 and mitophagy [93]. Exogenous antioxidant N-acetylcysteine can upregulate p53/TIGAR-mediated inhibition of Bnip3-dependent mitophagy, indicating that ROS signaling is essential for the p53/TIGAR/Bnip3-dependent mitophagy inhibition pathway [97]. In addition, p53 also induced damage-regulated autophagy regulator (DRAM) and Bax [93]. Studies have shown that p53/DRAM might promote autophagic apoptosis by upregulating mitophagy. Therefore, p53 is a regulator that balances the properties of mitophagy and could be an inhibitor that explicitly delays heart failure during the aging process and the TIGAR-represented glycolysis mechanism.

**Fig. 5:**
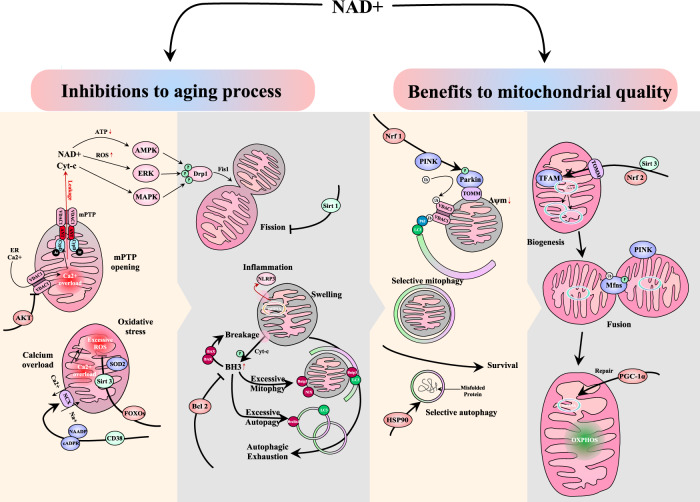
NAD+ associated mitochondrial protection in the aging heart. The presented figure showed that NAD+ relates two aspects of protection. The inhibitory role of NAD+ is involved in mitochondrial permeability, oxidative stress, calcium homeostasis, mitochondrial dynamics of fission, pre-apoptosis of BH-3 only domain protein, NLRP3-induced inflammations, and autophagic exhaustion. They are suppressed by AKT, FOXOs, CD38, Sirt1, Bcl-2, respectively. The assisting role of NAD+ is involved in Nrf1-induced selective mitophagy, Hsp90-mediated selective autophagy, Sirt3/Nrf2-mediated mitochondrial biogenesis, PINK1-induced fusion process, PGC-1-1α-dependent mitochondrial DNA repair, and NAD+ itself- dependent OXPHOS.

Studies have shown that overexpression of P49/strap in the aging heart led to reduced NAD+/NADH ratio, protein deacetylation induction, inhibition of PGC-1-induced mitochondrial fusion proteins mfn1/2 and Opa, and mitochondrial impairment [17]. Moreover, NAD+ also directly deacetylated Opa-1 to regulate its gene transcription, which further increased the ratio of Opa-1/VDAC1 and promoted the regulation of mitochondrial dynamics, leading to strengthened mitochondrial fusion and inhibition of mitochondrial senescence [98]. High expression of *p49* also inhibited the Sirtuin signal-related mitochondrial repair mechanism, closely related to *Sirt3, Sirt4*, and *Sirt5* expressions in mitochondria [49]. For example, in HUEVCs cells, Sirt4 regulates ATP homeostasis via AMPK and mitochondrial inner membrane ADP/ATP-translocase 2, which inhibited malonyl-CoA decarboxylase, and led to an increase in malonyl-CoA levels [99]. In addition, inhibited Sirt4 affects the transport of long-chain fatty acids to the mitochondrial matrix, blocking carnitine palmitoyltransferase 1 and inducing senescent lipid metabolism abnormalities [99]. This change further inhibited mitochondrial OXPHOS and induced glycolysis, causing storage of mitochondrial fatty acids with irreversible changes in morphology in the end [16]. With the increase in age, the quality and function of mitochondria decrease, resulting in the development of insulin resistance and metabolic diseases in the elderly. Moreover, Wnt signaling plays a vital role in the progression of heart disease, including decreased insulin sensitivity, cardiovascular remodeling, and structural changes, such as fibrosis, sclerosis, atherosclerosis, smooth muscle cell proliferation, and hypertrophy [100]. It is known that Wnt/β-catenin signaling has a developmental stage-specific biphasic effect on the cardiomyogenesis of mouse embryonic stem cells. Wnt signaling promotes cardiomyocyte differentiation at an early stage and inhibits it at a late stage [101]. Although the Wnt signal can affect myocardial mitochondrial damage through GSK-3β, it is also an intermediate bridge among insulin signal, angiotensin II (AngII) signal, and calcium signal, causing a reduction in the functional performance of the heart during the treatment of heart failure and arrhythmia [102]. However, there is no direct evidence of interaction between NAD+ and the Wnt/β-catenin pathway in the cardiovascular field of aging. Considering the typical role of Wnt signaling in anti-aging of multiorgan, NAD+-dependent deacetylase Sirt1 may also directly or indirectly regulate the epigenetic changes around the promoter region of the *LGR 5* gene which can be a novel concept for future research [103]. Figure 5 is a schematic diagram showing mechanisms of NAD+ associated mitochondrial protection.

In conclusion, NAD+ is a fundamental regulator of mitochondrial homeostasis, genomic stability, neuroprotection, healthy aging, and longevity. Studies have shown that WRN regulates the transcription of NMNAT1, a critical NAD+ biosynthetic enzyme [33]. At the organism level, NAD+ supplementation significantly prolongs the life span of *Caenorhabditis elegans* and *Drosophila melanogaster* affected by Werner Syndrome (WS) and delays animal aging and stem cell dysfunction. The accelerated senescence of WS is mediated by a metabolic disorder, impaired mitochondrial function, and mitochondrial phagocytosis. By maintaining an optimum level of NAD+, cells can respond to the WS phenotype, which provides a basis for NAD+ to simultaneously act in the maintenance of multiple organ functions, thereby benefiting the working environment of the heart.

## Therapeutic potential of targeting NAD+ in delaying heart aging

### Endogenous regulation

Clinical experiments have shown a high correlation between the reduction in blood NAD+ levels and heart failure in the elderly [104]. There are still some limitations in the existing interventions for heart aging. Some new interventional strategies used to prevent cardiovascular stresses via synthesis of more NAD+ have been established, such as ischemic preconditioning (IP), continuous low-to-medium intensity exercise, and other more exogenous drug strategies. For example, AngII mediated an increase in NADPH through JNK and p38^MAPK^ pathways. However, this also caused an increase in the mitochondrial ROS production [105]. A moderate increase in ROS could promote adaptability to IP-cardiac oxidative stress and mediate mitochondrial protective signaling [106]. Moreover, sevoflurane post-treatment could preserve NAD+ levels and inhibit mPTP opening in the aging myocardium under stress by increasing the expressions of PI3K and ERK1/2 pathways [107]. Exogenous mPTP inhibitor CsA, when interacting with CypD, prevented Ca^2+^-induced CypD from combining with ANT and inhibited Ca^2+^ mitochondrial output, which gave rise to decreased ΔΨm, and blocked ROS production. Moreover, CsA was used as a clinical drug to resist organ rejection. Its significant inhibitory effect on immune function makes it unsuitable for treating long-term chronic diseases, including cardiac hypertrophy and heart failure [11]. Additionally, IGF-1 could also activate Sirt1 regulation by phosphorylation of JNK1. Whether this pathway is dependent on the NAD+ signal remains to be further explored. Sirt1 KO caused oxidative stress damage, leading to an increase in the MDA production [40]. Furthermore, a precursor of NAD+, supplementation with NMN helps inhibit the aging-induced myocardial remodeling and heart failure and provides a new treatment strategy [72]. In a human study, NMN supplementation improved cardiac glycogen synthesis and gluconeogenesis in various subjects. With the increased level of mitochondrial function, PCr/ATP ratio and cardiac work capacity also increased [60]. In cardiomyocytes, the high NAD+ level may co-exist with the ATP/O2 coupling [19]. However, a high NADH level is also an important factor in triggering oxidative stress, enhancing SDH enzyme activity, and preserving PEP help to inhibit this process and improve the duration of NMN efficacy [39]. To optimize the intervention plan that can stimulate the downstream protective mechanism of NAD+ signaling, it is suggested that the properties of NMN utilization for anti-heart aging should be evaluated for the state of metabolism.

NAD+ is an important regulatory target of oxidative stress, whether in the form of oxygen or nitrogen radicals. The NAD+-related Sirt1 pathway acts on the deacetylation of eNOS and induces its activation, which helps reduce the harmful effects of ROS. It should be pointed out that in the absence of NAD+ signal induction, a higher eNOS level may damage cardiomyocytes [22]. Nonetheless, NO is thought to help inhibit hypertrophic cardiomyopathy by inducting cGMP pathways [71]. Recent studies have shown that NO-mediated cysteine oxidation may inhibit the glutathione by mitochondrial Sirt3 and Sirt5, thereby accelerating cardiomyocyte senescence and tissue inflammatory expression [108]. Other studies have shown that the upregulation of Sirt1 expression accompanied cell senescence induced by high glucose and high fat intake. Simultaneously, the aging of cardiomyocytes was inhibited through the AMPK pathway [61]. The main factor of cardiomyocytes senescence by high glucose and fat intake may be related to inhibiting mitochondrial adenosine transport [99]. Resveratrol administration revealed high Nrf2 induction ability in the concentration range of 1–20 μM, which led to the inhibition of high-glucose-induced apoptosis, increased amount of DNA fragments, and prolonged life span [109]. When combined with long-term low-intensity exercise, Resveratrol supplementation effectively increased NAD+ level and enhanced the activities of cardiac antioxidant enzymes, such as GSH, SOD, and CAT [110]. Similar biological effects were also demonstrated by natural active substances rich in polyphenols and oleic acid esters, etc [111, 112]. It should also be noted that some factors that regulate NAD+ levels may have different expressions based on specific cellular states. For example, Over-activated Sirt1 and Sirt4 could aggravate cardiac hypertrophy [113]. Zhu et al. [69] reported that isoflurane-induced drug preconditioning used to treat acute myocardial stress could not generate NAD+ benefits in elderly rats and was directly related to the mitochondrial damage caused by a decrease in GSK-3β phosphorylation. It is suggested that moderate precursor supplementation and normal mitochondrial function are important prerequisites for maintaining the effective action of NAD+ protection signaling on aging cardiomyocytes. Furthermore, the loss of NAD+ leads to the interruption of oxidative phosphorylation, which is also an important cause of age-related oxidative stress injury [69, 114].

### Strategy for choosing inhibitors

It is feasible to activate NAD+ expression by other endogenous stimuli to induce adaptive improvement and inhibit heart failure. For example, the energy restriction assists the NAD+-induced deacetylation of downstream p53 and NF-κB via Sirt2, leads to prolonged life span and improved anti-aging effect cardiomyocytes [49, 115]. Recent studies have shown that exercise can significantly increase the expression level of myocardial NAD+. Although the specific mechanism is still unclear, it may be related to ATP consumption leading to a higher AMPK level [116]. Conversely, the deletion of the NAD+ encoding gene can cause the loss of anti-heart failure effects, such as initially suppressed arrhythmia, systolic dysfunction, fibrosis, and compensatory heart rate [117]. An adequate supply of NAD+ in cardiomyocytes inhibits the transcription level of pro-inflammatory factors by reducing oxidative stress, decreasing the inflammatory response such as CD38, one of the NADases. Reduction in DNA oxidative stress damage, inactivation of PARP1, inhibition of NAD+ clearance pathway, and NAD+ synthesis can be achieved by reducing ROS [118]. Similarly, CD38 inhibitors, such as 78C, protect against cardiomyocyte senescence by preserving NAD+ [19]. In a previous study, continuous two weeks treatment with PARP1 blocker PJ-34 showed inhibition of endothelial cell aging, improvement in eNOS-mediated peripheral vascular protection, enhanced NAD+-dependent endothelial function, improved mitochondrial functional homeostasis and ROS inhibition [119]. Therefore, it is speculated that the various exogenous NAD+ precursor preservatives have similar protective effects on cardiac aging. However, further research is needed to verify this speculation [119]. In contrast, the NAMPT blocker FK866, an emerging tumor growth inhibitor, reduced cardiomyocyte stress tolerance [120]. Specifically, if we want to use NAD+ precursor supplement to prevent cardiotoxicity-related heart failure caused by drugs (e.g., Doxorubicin), more complex factors such as inflammation, tumors, metabolic abnormalities, neurodegeneration, and other diseases should be considered in advance. In other words, we cannot simply use, for instance, NMN to treat patients undergoing chemotherapy or similar treatments. For example, during tumor treatment with certain chemotherapeutics, where the NMN pathway needs to be blocked, it may be more feasible to use NAR instead of NMN [31].

### Targeting the inflammatory response

Since inflammation is one of the main features of heart aging, when exogenous stress is stimulated to induce adaptability, it is imperative to use the exogenous supplement to suppress the adverse effects of stress and reduce inflammation-induced senescence (SASP) [56]. The senescence of cardiomyocytes significantly increased the expression of TNF-α and NF-κB. TNF-α caused mitochondrial mPTP opening, apoptosis, and downregulation of phosphatase and tensin homolog signal, resulting in excessive Bnip3-dependent mitophagy and mitochondrial dysfunction [79, 80]. Further, TNF-α decreased the level of NAD+ and produced ROS by inducing an increase in NAD(P)H oxidase expression [65]. TNF-α also caused an increase in *CD38* expression and the conversion of NAD+ to NAADP and CADPR. By acting as a regulator, TNF-α increases calcium release from the cardiac endoplasmic reticulum and causes intracellular calcium overload. Higher adrenaline sensitivity exacerbates the cardiac burden and the risk of mitochondrial injury [121]. As for the downstream mechanism, due to the inhibition of NAD+/Sirt pathway, downregulation of Sirt1 causes decreased expression of *FOXO, p53, PPARα*, and *Akt* in the nucleus; reduced transcription levels of antioxidant enzymes, such as *Bcl-xL*; increased levels of intracellular oxidative stress; decreased autophagy; decreased fatty acid oxidation capacity; and increased level of apoptosis. On the other hand, the expression of *NLRP3* inflammasome in myocardial tissue is necessary for the NAD+/Sirt1-mediated anti-aging mechanism [57]. However, some studies have suggested using small molecule drugs to inhibit the formation of NLRP3 complex that helps inhibit myocardial fibrosis, dilated cardiomyopathy, and atherosclerosis [56]. Therefore, NAD+ might be a transformer that affects the endogenous effects of NLPR3, and appropriate NAD+ supplementation could reduce myocardial inflammation using activated SIRTs and NRF2 signaling (Table 1).

**Table 1:** NAD+ is involved in the mechanistic pathways underlying the cardioprotective effects of different interventions to cardiac dysfunction.

Intervention	Approach	Change in NAD+	Outcome	Benefits of NAD+?	Reference
AngII	I/R in aged Wistar rats	NAD+/NADH increased by about 7.4%	AngII inhibits mPTP and cardiolipin Peroxidation opening, which exerted a noticeable cardioprotective effect on old rats.	Yes	[76]
Sevoflurane	I/R in aged SD rats	An increase of about 153.46%	The Sevoflurane-mediated cardio-protection in young rats was not effective in aging rats, which may result from failure to activate Akt and ERK1/2 and failure to inhibit mPTP opening.	N/A	[107]
NMN	Wild type C57BL/6N old mice	An increase of about 29.66%	NMN effectively reduced age-related physiological decline in mice.	Yes	[122]
isoflurane	I/R in Fischer-344 rats	An increase of about 103.51%	Isoflurane pretreatment inhibited the opening of mPTP. The I/R damage to the mitochondria of young mice was improved in all aspects of morphological characteristics. But no effect on aged mice.	N/A	[69]
CD38 inhibitor: 78C	Aged C57BL/6 mice	An increase of about 38.46%	78C treatment decreased ejection fraction and fractional shortening rate; improved systolic left ventricular volume and isovolumic contraction, and restored NAD+ in aged myocardium to the level of young mice	Yes	[19]
NAMPT blocker FK866	Cardiomyocytes of neonatal SD rats	Reduced by about 70%	Fk866 reduced NAD+ level by inhibiting NAMPT activity and impaired mitochondrial metabolism and adaptive response of cardiomyocytes to norepinephrine, hydrogen peroxide, and insulin.	Yes	[120]
NAR+ FK866	CIPN model	An increase of about 500%	NRK1/NRK2 phosphorylation produced NAMN, which further promoted NAD+ synthesis.	N/A	[31]
exercise	HFD model of w^1118^ Drosophila	An increase of about 25.16%	Exercise training reduced lipid accumulation, enhanced cardiac function, activated the NAD+/dSIR2/PGC-1α pathway, and reduced the risk of arrhythmia, thereby improving lipotoxic cardiomyopathy induced by cardiac dsir 2-RNAi.	Yes	[116]
Agomelatine	I/R in Wistar rats	An increase of about 36.76%	Agomelatine inhibited the opening of mPTP, significantly improved cardiac function, reduced the pathological changes of ischemic myocardium, and reduced the area of myocardial infarction.	Yes	[123]
DAPH	Wistar rats	Reduced by about 56%	DAHP accelerated cell senescence and inhibited GTPCH1, and reduced the level of NAD+	Yes	[124]
Tilianin	I/R in aged SD rats	An increase of about 79.14%	Tilianin regulates the AMPK / SIRT1 / PGC-1a signaling pathway, which can significantly reduce myocardial infarction, improve the histopathological morphology of ischemia-reperfusion myocardium, and reduce oxidative stress injury regulates energy metabolism disorder.	Yes	[125]
PARP inhibitor: 3-AB	Sepsis model of cecal ligation and perforation in SD rats	An increase of about 91.3%	3AB reduced myocardial cell mitochondrial cristae breakage and myofibril breakage, which indicated that 3AB plays a protective role in edema or structural disorders and inhibits PARP activation.	Yes	[126]
Nigella sativa	I/R in Wistar rats	An increase of about 168.18%	Inhibiting the opening of MPTP can significantly restore cardiac function after ischemia-reperfusion.	Yes	[127]
NR	Healthy human	An increase of about 173.59%	Inhibition of mitochondrial dysfunction with elevated NAD+ level in the blood.	Yes	[128]

The majority demonstrated the benefit of increasing NAD+ in the aging heart.

## Conclusions

NAD+ depletion is one of the primary pathogenesis of heart disease, and NAD+ supplementation has shown therapeutic potential for restoring healthy metabolism and physiological functions. The pluripotency of this abundant coenzyme NAD+ facilitates its availability for exerting a therapeutic effect. In particular, NAD+ acts as an auxiliary substrate in the deacylation reaction carried out by multiple interactions between nucleus transcription signaling in the heart. These NAD+-dependent signals are associated with or directly control several aspects of metabolism, especially in the mitochondria. A large amount of data indicated that supplementing NAD+ to enhance multiple intercellular cross-talks may be a feasible treatment option for inhibiting overall body aging. Simultaneously, it should be considered that the heart, as a blood-pumping organ, is highly dependent on the metabolic environment and ion homeostasis. Notably, both suitable doses and the form of NAD+ precursor supplements are crucial for gaining their optimum benefits. NAD+ supplementation should be used with caution in certain circumstances, such as myocardial hypoxia and hypertrophy. Further studies are needed to elucidate a closer connection between multiple cardioprotective signals and NAD+, which will help to improve the accuracy and effectiveness of interventions on heart aging.

## Data Availability

The datasets generated during and analyzed during the current study are available from the corresponding author on reasonable request.
